# Impact of a cumulative positive fluid balance during the first three ICU days in patients with sepsis: a propensity score-matched cohort study

**DOI:** 10.1186/s13613-023-01178-x

**Published:** 2023-10-19

**Authors:** Dong-gon Hyun, Jee Hwan Ahn, Jin Won Huh, Sang-Bum Hong, Younsuck Koh, Dong Kyu Oh, Su Yeon Lee, Mi Hyeon Park, Haein Lee, Chae-Man Lim, the Korean Sepsis Alliance (KSA) Investigators, Dong Kyu oh, Gee Young Suh, Kyeongman Jeon, Ryoung-Eun Ko, Young-Jae Cho, Yeon Joo Lee, Sung Yoon Lim, Sunghoon Park, Jeongwon Heo, Jae-myeong Lee, Kyung Chan Kim, Yeon Joo Lee, Youjin Chang, Kyeongman Jeon, Sang-Min Lee, Suk-Kyung Hong, Woo Hyun Cho, Sang Hyun Kwak, Heung Bum Lee, Jong-Joon Ahn, Gil Myeong Seong, Song-I Lee, Sunghoon Park, Tai Sun Park, Su Hwan Lee, Eun Young Choi, Jae Young Moon, Hyung Koo Kang

**Affiliations:** 1 Department of Pulmonary and Critical Care Medicine, Asan Medical Center, University of Ulsan College of Medicine, 88 Olympic-Ro 43-Gil, Songpa-Gu, Seoul, 05505 Republic of Korea; 2 Samsung medical center, seoul, South Korea; 3 Seoul National University Bundang Hospital, seongnam, South Korea; 4 Hallym University Sacred Heart Hospital, Anyang, South Korea; 5 Kangwon national university hospital, Gangneung, South Korea; 6 Korea University Anam Hospital, Seoul, South Korea; 7 Daegu Catholic University Hospital, Daegu, South Korea; 8 Inje University Sanggye Paik Hospital, Seoul, South Korea; 9 Seoul National University Hospital, Seoul, South Korea; 10 Pusan National University Yangsan Hospital, Pusan, South Korea; 11 Chonnam National University Hospital, Gwangju, South Korea; 12 Jeonbuk National University Hospital, Jeonju, South Korea; 13 Ulsan University Hospital, Ulsan, South Korea; 14 Jeju National University Hospital, Jeju, South Korea; 15 Chungnam National University Hospital, Daejeon, South Korea; 16 Hanyang University Guri Hospital, Guri, South Korea; 17 Severance Hospital, Seoul, South Korea; 18 Yeungnam University Medical Center, Daegu, South Korea; 19 Chungnam National University Sejong Hospital, Sejong, South Korea; 20 Inje University Ilsan Paik Hospital, Ilsan, South Korea

**Keywords:** Sepsis, Septic shock, Resuscitation, Mortality, Fluid therapy

## Abstract

**Background:**

The optimal strategy for fluid management during the first few days of ICU in sepsis patients remains controversial. We aimed to investigate the impact of cumulative fluid balance during the first three days of ICU on the mortality of patients with sepsis.

**Methods:**

This study analyzed prospectively collected data from the Korean Sepsis Alliance Database, which registered 11,981 sepsis patients from 20 hospitals. We selected three propensity score-matched cohorts consisting of patients with a negative or positive cumulative fluid balance during the first three ICU days: from ICU admission to the first midnight as the D1 cohort, until the second midnight as the D2 cohort, and until the third midnight as the D3 cohort. The propensity score for fluid balance was calculated using covariates including the amount of fluid output during the first three ICU days. The primary outcome was mortality at day 28 in the ICU.

**Results:**

From a total of 11,981 patients, 2516 patients were included for propensity score matching. After matching in a 1:1 ratio, there were 483, 373, and 392 matched pairs of patients assigned to the D1, D2, and D3 cohorts, respectively. In the D1 cohort, there were no significant differences in mortality at day 28 (hazard ratio [HR], 1.17; 95% confidence interval [CI] 0.85–1.60; *P* = 0.354) between the two groups. The positive fluid groups in both the D2 (HR, 2.13; 95% CI 1.48–3.06; *P* < 0.001) and D3 (HR, 1.56; 95% CI 1.10–2.22; *P* = 0.012) cohorts had significantly higher mortality rates than the negative fluid groups.

**Conclusions:**

In patients with sepsis, a positive fluid balance on the first ICU day was not associated with mortality at day 28. In contrast, cumulative positive fluid balances on the second and third ICU days were associated with higher mortality at day 28.

**Supplementary Information:**

The online version contains supplementary material available at 10.1186/s13613-023-01178-x.

## Background

The time-dependent bundle has recently become a key strategy for the treatment of sepsis [1, 2]. Since the landmark development of Early Goal-Directed Therapy, fluid resuscitation has been an essential component of the sepsis bundles used by clinicians [3, 4]. Initial fluid resuscitation can increase the stroke volume and restore organ perfusion in some cases of patients with sepsis-related hypovolemia induced by generalized vasodilation [5]. The Surviving Sepsis Campaign recommends at least a 30 mL/kg (ideal body weight) level of intravenous crystalloids within the first 3 h of initial resuscitation [6]. However, there have been growing concerns that excess fluid accumulation may be harmful to patients with sepsis [7–9]. Whereas the hemodynamic benefits of preload optimization after a fluid bolus are maintained for less than an hour, excess fluids during sepsis may damage vascular integrity leading to tissue edema with concurrent organ dysfunction [10–13]. Previous clinical trials of sepsis resuscitation via the administration of fluids have not demonstrated an improvement in clinical outcomes [14–17]. Moreover, recent studies focusing on a fluid restriction strategy after initial resuscitation did not show a lower mortality rate compared with a liberal fluid strategy [18, 19]. The optimal fluid management approach for sepsis following an initial fluid resuscitation thus remains unresolved [20].

The influence of a positive fluid balance after the initial fluid resuscitation on survival has been evaluated in several prior studies. A previous pan-European study reported that a positive fluid balance within the first 72 h was the strongest prognostic indicator of death in patients with intensive care unit (ICU)-acquired sepsis [21]. Furthermore, it has been reported in a single-center study that patients with severe sepsis or septic shock who had a greater than 2.5 L fluid balance at 72 h had a higher mortality rate [22]. Notably however, conflicting results have been reported for the optimal cumulative fluid balance target after the initial resuscitation. A large, observational, cohort study has reported that the administration of more than 5 L of fluid during the first ICU day was associated with an increased risk of death [23]. On the other hand, another international observational cohort study indicated that the cumulative fluid balance at day 3 after ICU admission, but not in the first 24 h, was independently associated with a higher 28-day mortality [24]. Given these findings, we aimed to investigate the impact of cumulative fluid balances during the first three days in the ICU on the mortality rate of patients with sepsis. We therefore designed a propensity-score matching study to compare the prognosis of patients with a positive fluid balance and those with a negative fluid balance using three cohorts with varying time windows from ICU admission (e.g., until the midnight of day 1 vs. day 2 vs. day 3).

## Methods

### Data source and patient selection

We analyzed prospectively collected data of 11,981 patients with sepsis from 20 tertiary referral or university-affiliated hospitals in South Korea between September 2019 and December 2021 as part of an ongoing nationwide, multicenter observational cohort (the Korean Sepsis Alliance registry). The protocols for patient enrollment and data collection have been described previously [25]. All consecutive patients who presented to the emergency department or hospitalized patients in wards who were managed by a rapid response team were screened for eligibility, and patients aged ≥ 19 years diagnosed with sepsis based on the third International Consensus Definitions for Sepsis and Septic Shock (Sepsis-3) were included in the registry [26]. We selected adult patients (age ≥ 19 years) who had been admitted to and remained in an ICU for at least three days. Patients who did not have recorded fluid data within these first three days of ICU management were excluded from further analysis.

### Time window and cumulative fluid balance

**Fig. 1:**
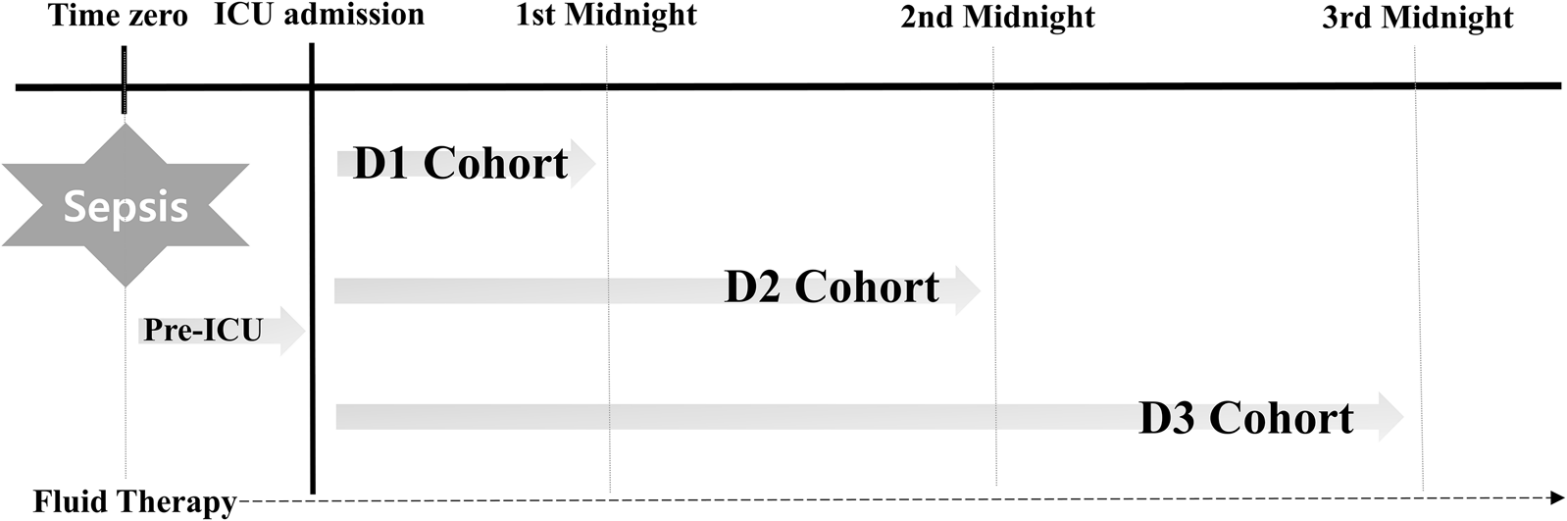
Schematic representation of timeline during the first three ICU days. Time zero was defined as the time a patient first entered the emergency room or the time of screening by the rapid response team. We defined D1 as the time from ICU admission to the first midnight, D2 as the time from ICU admission to the midnight of the second ICU day, and D3 as the time from ICU admission to the midnight of the third ICU day

In this study, each “time window” was defined as follows: “time zero” referred to the time a patient first entered the emergency room (for those patients admitted to ICU through the emergency room) or the time of screening by the rapid response team (for those patients admitted to the ICU from a ward). The “time before ICU admission” referred to the interval from the time immediately prior to before ICU admission. We defined D1 as the time from ICU admission to the first midnight, D2 as the time from ICU admission to the midnight of the second ICU day, and D3 as the time from ICU admission to the midnight of the third ICU day (Fig. 1). Fluid input included both enteral and parenteral intake, and fluid output consisted of urinary output, drainage fluid, and extracorporeal fluid elimination, but not insensible fluid loss. In addition, we separately evaluated the fluid inputs and outputs prior to ICU admission. Since the amounts of fluid were recorded only by each hospitalization day, the fluid balance, i.e., net, was calculated based on the daily fluid input and output. The cumulative fluid balances of D1, D2, and D3 were calculated as follows:: $\sum_{x=1}^{n}Ix - \sum_{x=1}^{n}Ox$ (*I* = the amount of fluid input at ICU day *n*, *O* = the amount of fluid output at ICU Day *n*, *n* = ICU Day).

### Propensity score matching and outcomes

To adjust for any confounding variables in our comparisons of outcomes between the two groups, we identified three propensity score-matched D1, D2, and D3 cohorts from the entire unmatched cohort. The propensity score for the cumulative fluid balance was calculated for each cohort using a multivariable logistic regression model to estimate the probability of a cumulative positive fluid balance at D1, D2, or D3. The models included covariates that may affect the likelihood of fluid balance: age, gender, sequential organ failure assessment (SOFA) score, the presence of septic shock, referring facility, comorbidity, site of infection, adjunct interventions, microbiologic pathogen, and the amount of fluid output [26]. Covariables such as referring facility, comorbidity, and site of infection were based on the previous study [27]. For example, chronic liver disease was defined as a prolonged course of hepatic disease for more than 6 months, excluding hepatocellular cancer. Adjuvant interventions included steroid therapy, mechanical ventilation, continuous renal replacement therapy, extracorporeal membrane oxygenation, or vasopressor at ICU admission. Only those microorganisms detected via culture, serology, or molecular or histological investigations within 48 h of time zero and deemed to be true pathogens were adjusted. Matching was performed using a 1:1 nearest-neighbor algorithm without replacement and with a caliper width of 0.1. To evaluate the balance between the groups before and after matching, absolute standardized mean differences (SMDs) were calculated, with values < 0.1 used to indicate a lack of any meaningful imbalance [28]. In addition, we used c-statistics to assess the goodness of fit for the fluid balance in the model.

Patients were divided into a negative or positive fluid balance group in the three matched D1, D2, and D3 cohorts. We compared the mortality rates at 28 days post ICU admission between the negative and positive fluid balance groups in each cohort. Secondary outcomes included the proportions of patients with freedom of mechanical ventilation or that had been discharged from the ICU at 28 days.

### Statistical analysis

Data are presented as numbers with percentages for categorical variables and means plus standard deviations or medians with interquartile ranges (IQR) for continuous variables. A linear mixed model was used to compare continuous variables with the matched cohorts. For time-to-event analysis, the Kaplan–Meier method was used to estimate survival curves and cumulative incidence curves were generated for both the time to discharge alive from the ICU and the time to freedom from invasive ventilation. Time-to-event analysis was right-censored at 28 days. The primary outcome of death within 28 days of ICU admission between the two groups was compared using clustered Cox proportional hazards regression analysis. The results are presented as a hazard ratio (HR) with a 95% confidence interval (CI). The proportional hazard assumption was assessed through an inspection of Schoenfeld residuals. Secondary outcomes were evaluated via competing-risks regression based on a clustered Fine and Gray’s proportional subhazards model. Death before day 28 was considered to be the competing event. This analysis provided sub-hazard ratios (sHRs) and 95% CIs. Two-sided *P* values < 0.05 were considered to indicate significance. All analyses were performed using R software version 4.1.2 (R Core Team).

## Results

### Study cohort and baseline characteristics

A total of 11,981 patients with a diagnosis of sepsis were admitted to the 20 participating hospitals during the study period (Additional file 1: Fig. S1). Among these cases, 9465 patients who were younger than 19 years old, not admitted to an ICU, stayed for less than 72 h in an ICU, or had missing fluid data during the 3 days of ICU management were excluded. The final cohort analyzed in this study thus comprised 2516 patients, and their baseline characteristics are presented in Additional file 1: Table S1. The median age of the study population was 74.0 years (IQR, 64.0–81.0) and 1066 (42.3%) were female. The percentage of patients who received fluid resuscitation for sepsis management was 83.1%. The median SOFA score was 7.0 (IQR, 5.0–9.0) and the most common comorbidity was diabetes mellitus (39.1%), followed by neurologic disease (28.5%). The main type of infection was respiratory infection (48.3%). In terms of intervention, 561 (22.3%) received steroids and 1274 (50.6%) required mechanical ventilation. Microbial pathogens were detected in 1665 patients (66.2%), with 673 of these cases (46.3%) presenting with multi-drug-resistant bacterial infections. The overall mortality rate at day 28 was 23.4% (n = 588). The median length of the hospital stay before ICU admission and total hospitalization time was 0.0 days and 16.0 days (10.0–27.0), respectively.

### PS-matched cohorts

Among the entire cohort of 2,516 patients, 483, 373, and 392 pairs were matched using PS score at a 1:1 ratio to the D1, D2, and D3 cohort, respectively. The C-statistic was 0.749 (95% CI, 0.727–0.771) in the D1 model, 0.844 (95% CI, 0.826–0.861) in the D2 model, and 0.836 (95% CI, 0.819–0.854) in the D3 model. The distributions of the SMDs for matched variables revealed that almost all of the patients were closely matched, as indicated by an improvement in the balance after matching (Additional file 1: Fig. S2). The baseline characteristics and balance statistics of the three matched cohorts are listed in Table 1. Compared to those in the entire unmatched cohort, patients in the matched cohorts less commonly received fluid resuscitation, steroids, mechanical ventilation, or vasopressors.

**Table 1:** Baseline characteristics of the propensity score matched cohorts stratified by the Day 1–3 fluid balances

Characteristic	Day 1 cohort (n = 966)		SMD	Day 2 cohort (n = 746)		SMD	Day 3 cohort (n = 784)		SMD
	Negative (n = 483)	Positive (n = 483)		Negative (n = 373)	Positive (n = 373)		Negative (n = 392)	Positive (n = 392)	
Female, %	201 (41.6)	188 (38.9)	0.055	154 (41.3)	145 (38.9)	0.049	163 (41.6)	158 (40.3)	0.026
Age, yr	73.00 [63.00, 81.00]	74.00 [63.00, 80.00]	0.006	72.00 [62.00, 81.00]	73.00 [63.00, 80.00]	0.033	73.00 [63.00, 81.00]	72.50 [63.00, 80.00]	0.047
BMI, kg/m^2a^	22.04 [19.24, 25.06]	21.80 [19.35, 24.41]	0.099	22.16 [19.54, 24.93]	22.2 [19.60, 24.77]	0.04	21.81 [19.06, 24.85]	22.03 [19.55, 24.49]	0.052
Resuscitative fluid, %	376 (77.8)	383 (79.3)	0.035	287 (76.9)	277 (74.3)	0.062	298 (76.0)	308 (78.6)	0.061
Severity scores									
SOFA score	7.00 [5.00, 9.00]	6.00 [5.00, 8.00]	0.068	7.00 [5.00, 9.00]	6.00 [5.00, 9.00]	0.043	7.00 [5.00, 9.00]	7.00 [5.00, 9.00]	0.027
Septic shock, %	101 (20.9)	93 (19.3)	0.041	82 (22.0)	83 (22.3)	0.006	84 (21.4)	87 (22.2)	0.019
Referring facility, %			0.043			0.07			0.049
Community	326 (67.5)	323 (66.9)		257 (68.9)	262 (70.2)		261 (66.6)	265 (67.6)	
Health care	35 (7.2)	31 (6.4)		16 (4.3)	20 (5.4)		20 (5.1)	23 (5.9)	
Hospital	122 (25.3)	129 (26.7)		100 (26.8)	91 (24.4)		111 (28.3)	104 (26.5)	
Comorbidities, %									
Cardiac	128 (26.5)	136 (28.2)	0.037	109 (29.2)	109 (29.2)	< 0.001	111 (28.3)	111 (28.3)	< 0.001
Lung	73 (15.1)	71 (14.7)	0.012	53 (14.2)	60 (16.1)	0.052	58 (14.8)	63 (16.1)	0.035
Neurologic	140 (29.0)	148 (30.6)	0.036	92 (24.7)	90 (24.1)	0.012	103 (26.3)	96 (24.5)	0.041
Liver	47 (9.7)	43 (8.9)	0.028	33 (8.8)	27 (7.2)	0.059	36 (9.2)	38 (9.7)	0.017
Diabetes mellitus	190 (39.3)	193 (40.0)	0.013	161 (43.2)	163 (43.7)	0.011	160 (40.8)	177 (45.2)	0.088
Renal disease	67 (13.9)	61 (12.6)	0.037	64 (17.2)	73 (19.6)	0.062	71 (18.1)	67 (17.1)	0.027
Connective tissue disease	10 (2.1)	10 (2.1)	< 0.001	15 (4.0)	11 (2.9)	0.058	11 (2.8)	13 (3.3)	0.03
Immunocompromised	15 (3.1)	9 ( 1.9)	0.08	11 (2.9)	5 (1.3)	0.111	15 (3.8)	6 (1.5)	0.143
Hematologic malignancy	16 (3.3)	16 (3.3)	< 0.001	9 (2.4)	9 (2.4)	< 0.001	11 (2.8)	12 (3.1)	0.015
Solid cancer	119 (24.6)	105 (21.7)	0.069	91 (24.4)	101 (27.1)	0.061	91 (23.2)	86 (21.9)	0.031
Site of infection, %									
Respiratory	232 (48.0)	226 (46.8)	0.025	184 (49.3)	194 (52.0)	0.054	196 (50.0)	200 (51.0)	0.02
Abdominal	106 (21.9)	107 (22.2)	0.005	81 (21.7)	81 (21.7)	< 0.001	91 (23.2)	83 (21.2)	0.049
Urinary tract	118 (24.4)	115 (23.8)	0.015	80 (21.4)	82 (22.0)	0.013	89 (22.7)	85 (21.7)	0.025
Others^b^	62 (12.8)	69 (14.3)	0.042	51 (13.7)	40 (10.7)	0.09	49 (12.5)	48 (12.2)	0.008
Laboratory findings									
White blood cell count * 10^3^/L	12.20 [8.21, 16.78]	12.02 [8.30, 18.27]	0.084	11.40 [7.46, 16.20]	12.21 [7.30, 18.00]	0.11	11.90 [7.80, 16.80]	12.52 [7.47, 17.60]	0.021
C-reactive protein, mg/dL	11.43 [3.34, 20.45]	11.70 [2.89, 21.66]	0.033	11.47 [2.72, 20.95]	12.70 [2.30, 21.65]	0.005	11.9 [3.35, 20.84]	12.92 [3.88, 22.27]	0.071
Procalcitonin^a^, ng/mL	2.93 [0.32, 34.82]	8.20 [1.22, 34.82]	0.025	3.96 [0.36, 34.89]	6.78 [1.03, 35.01]	0.028	4.96 [0.42, 31.81]	5.62 [1.05, 29.77]	0.011
Lactic acid^a^, mmol/L	2.60 [1.50, 4.54]	2.78 [1.70, 5.00]	0.087	2.70 [1.60, 4.77]	3.00 [1.70, 5.28]	0.132	2.67 [1.50, 4.56]	2.90 [1.70, 5.30]	0.182
Adjunct interventions, %									
Steroids	74 (15.3)	67 (13.9)	0.041	68 (18.2)	74 (19.8)	0.041	68 (17.3)	67 (17.1)	0.007
Mechanical ventilation	199 (41.2)	179 (37.1)	0.085	163 (43.7)	167 (44.8)	0.022	178 (45.4)	170 (43.4)	0.041
CRRT	64 (13.3)	51 (10.6)	0.083	60 (16.1)	68 (18.2)	0.057	67 (17.1)	62 (15.8)	0.034
ECMO	4 (0.8)	2 (0.4)	0.053	3 (0.8)	6 (1.6)	0.074	2 (0.5)	6 (1.5)	0.102
Vasopressors	337 (69.8)	347 (71.8)	0.046	269 (72.1)	261 (70.0)	0.047	281 (71.7)	278 (70.9)	0.017
Microbiologic pathogen, %	300 (62.1)	302 (62.5)	0.009	221 (59.2)	218 (58.4)	0.016	226 (57.7)	231 (58.9)	0.026
Bacteria	285 (95.0)	284 (94.0)	0.042	208 (94.1)	198 (90.8)	0.125	216 (95.6)	209 (90.5)	0.201
Virus	10 (3.3)	4 (1.3)	0.133	10 (4.5)	6 (2.8)	0.095	7 (3.1)	5 (2.2)	0.058
Fungus	13 (4.3)	23 (7.6)	0.139	10 (4.5)	20 (9.2)	0.185	9 (4.0)	22 (9.5)	0.222
MDR^a^	138 (51.3)	112 (42.4)	0.179	92 (46.7)	76 (41.5)	0.104	91 (45.3)	79 (40.5)	0.096

*BMI* body mass index, *SOFA* sequential organ failure assessment, *CRRT* continuous renal replacement therapy, *ECMO* extracorporeal membrane oxygenation, *MDR* multi-drug resistance

^a^The numbers of patients who had BMI measurements taken in the Day 1–3 cohorts were 942, 728, and 755, respectively. For procalcitonin, these were 506, 367, and 400. For lactic acid, 956, 735, and 773. For MDR, 533, 380, and 396

^b^Others included skin/soft tissue infection, catheter-associated infection, neurologic infection, and unknown

### Fluid balance

**Fig. 2:**
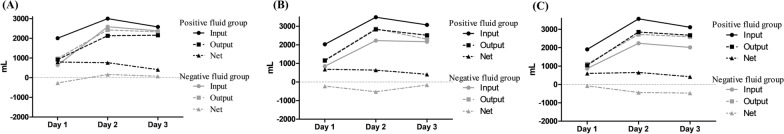
Daily median fluid intake, output, and net in matched cohorts over the first three days of ICU management on the positive and negative balance groups, stratified by fluid balances on each day. Day 1 cohort (**A**), Day 2 cohort (**B**), and Day 3 cohort (**C**)

In the entire unmatched cohort, the cumulative fluid balance during the first three days in the ICU was 1927.8 mL (IQR, 298.4–3835.9; Additional file 1: Table S2); moreover, fluid balance before ICU admission and daily fluid balance during the first three days of ICU were positive. In the D1 cohort, the difference in fluid output was not significant (*P* = 0.3) between the two groups, whereas the fluid input was higher in the positive fluid balance group (*P* < 0.001; Fig. 2). However, the negative fluid balance group in the D1 cohort had a positive net fluid amount in ICU day 2 (160.0 mL; IQR, − 573.5 to 843.5) and ICU day 3 (640.0 mL; IQR, 280.1 to 1204.6). In the D2 cohort, the fluid output between the positive and negative fluid balance groups was similar, except ICU day 3 (2510.0 mL vs. 2300.0 mL, *P* = 0.005). The net fluid amount remained consistently negative during the first three days in the negative fluid balance group. In the D3 cohort, the fluid output during the first three days was similar between the two groups, while patients in the negative fluid balance group had a lower fluid intake compared with those in the positive fluid balance group, leading to a continuous negative net fluid amount.

## Outcomes

**Fig. 3:**
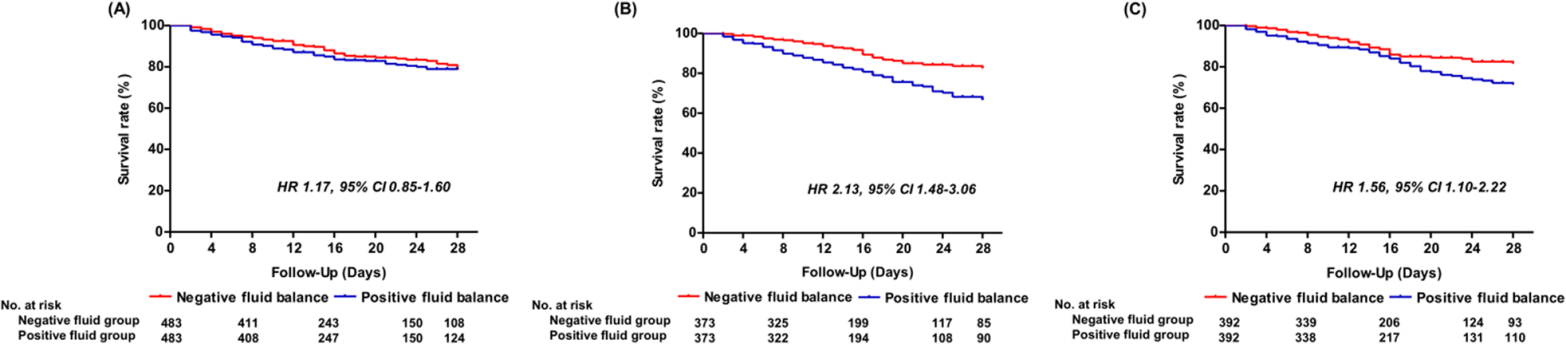
Kaplan–Meier estimates of the probability of survival to Day 28 in matched cohorts stratified by the fluid balances on Day 1 (**A**), Day 2 (**B**), and Day 3 (**C**). HR, hazard ratio; CI, confidence interval

**Fig. 4:**
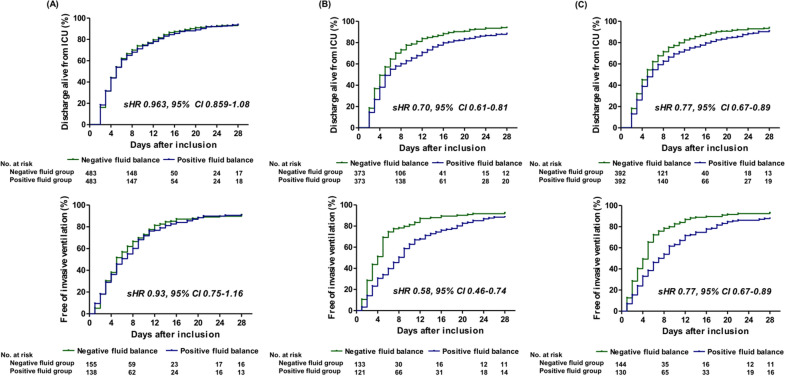
Cumulative ICU discharge and freedom of mechanical ventilation rates by Day 28 post ICU admittance in matched cohorts stratified by the fluid balances on Day 1 (**A**), Day 2 (**B**), and Day 3 (**C**). sHR, subhazard ratio; CI, confidence interval

In the D1 cohort, there was no significant difference in mortality at day 28 (HR, 1.17; 95% CI 0.85–1.60; *P* = 0.354) between the two groups (Fig. 3). In contrast, the mortality at day 28 was higher in the positive fluid group in both the D2 (HR, 2.13; 95% CI 1.48–3.06; *P* < 0.001) and D3 (HR 1.56; 95% CI 1.10–2.22; *P* = 0.012) cohorts. In addition, there was a stepwise increase in the death hazard at day 28 with increasing cumulative fluid balance in both D2 and D3 cohorts (Additional file 1: Table S3). With regard to secondary outcomes, similar results to the primary outcomes were observed in the three matched cohorts (Fig. 4). There were no differences in the freedom from mechanical ventilation (sHR, 0.93; 95% CI 0.75–1.16; *P* = 0.53) and ICU discharge at day 28 (sHR, 0.96; 95% CI 0.86–1.08; *P* = 0.51) between the two groups in the D1 cohort. The positive fluid cases in both the D2 (sHR, 0.70; 95% CI 0.61–0.81; *P* < 0.001) and D3 (sHR, 0.77; 95% CI 0.67–0.89; *P* < 0.001) cohorts were less likely to be discharged from the ICU than the patients in the negative fluid group. Additionally, freedom from mechanical ventilation (i.e., the rate of extubation) was consistently lower among patients in the positive fluid balance group in both the D2 cohort (sHR, 0.58; 95% CI 0.46–0.74; *P* < 0.001) and the D3 cohort (sHR, 0.77; 95% CI 0.67–0.89; *P* = 0.002).

## Discussion

We have analyzed three propensity score-matched cohorts of sepsis patients to examine the varying effects of fluid balance during the first three days of ICU. Our findings indicate that while a positive fluid balance on the first ICU day did not impact the 28-day mortality, a positive fluid balance that continued onto the second or third ICU day was associated with a higher rate of death by day 28 when compared with patients showing a negative fluid balance. For D2 and D3 cohorts, similar trends were observed for secondary outcomes (e.g., discharge from the ICU and freedom from mechanical ventilation). Taken together, our results show that cumulative positive fluid balances on the second and third ICU days were associated with increased mortality in patients with sepsis.

Numerous studies have been conducted in an attempt to develop an optimal fluid management strategy in the treatment of sepsis that will enhance survival outcomes. Given that higher fluid volumes have been associated with harm in a number of studies of sepsis cases, a prior meta-analysis was conducted of 11 randomized trials in critically ill patients that evaluated the benefits of conservative or deresuscitative strategies (active removal of fluid) and reported no significant effects on mortality between these two approaches [29]. Another systematic review has assessed the benefits and harms of lower versus higher fluid volumes in adult patients with sepsis but found no statistically significant differences in the all-cause mortality outcomes [30]. Recently, international, randomized trials involving ICU patients with septic shock also reported no significant differences in 90-day mortalities among the patients who received restricted fluid therapy and those who received standard therapy [18, 19]. On the other hand, there has been growing evidence of possible associations between positive fluid balance in sepsis patients after the initial resuscitation and eventual clinical outcome [21–24]. Our present study expands on these prior reports, and our results suggest that a positive fluid balance continued on ICU days 2 and 3 is associated with an increased risk of death in septic patients. Of note, a cumulative positive fluid balance on the second ICU day was associated with the highest risk for mortality.

Our current findings are also pertinent to the current debates about the timing of the fluid balance in sepsis [22, 24, 31]. Our results support the evidence from small retrospective studies reporting on the association between a higher accumulated positive fluid balance after the first 24 h and patient death [22, 31]. In addition, the dose–response relationship between the amount of fluid overload and mortality risk of our study is consistent with the result of a large, international cohort study that showed a stepwise increase in the risk of death with increasing 3-day cumulative fluid balance [24].

The mechanisms of positive fluid balance related to adverse outcomes in sepsis patients remain unclear. However, a conceptual model recommended previously may be of some help in explaining this [32]. In the four dynamic volume stages of resuscitation (rescue, optimization, stabilization, and de-escalation), sepsis patients in a compensated state after an initial rescue may enter an optimization stage with the aim of improving tissue perfusion and mitigating organ dysfunction. Another previous study evaluating daily SOFA score kinetics according to the fluid overload status demonstrated that the differences in the daily SOFA score according to the fluid overload occurred from day 3 and the changes in this value were also higher in the patients without fluid overload or having one day of fluid overload compared to those with two or more days of fluid overload [33]. Consequently, hypervolemia after the rescue phase of ICU on day 1 might exacerbate capillary leak into organs, thus contributing to organ dysfunction and subsequent organ failure.

There were several advantages to our current study over prior investigations. First, we examined the respective effects of the daily fluid balance on each of the first 3 days of ICU care, which was not done in previous studies. Second, our propensity-based analysis of a large prospective database involved well-balanced covariates, thereby allowing for the most reliable comparison of fluid balance effects. For example, comorbidities such as chronic kidney disease, which may have influenced clinical outcomes, were balanced between the two groups. Third, whereas prior studies analyzed the effect of net fluid balance without considering the profile of fluid output, we matched cohorts with similar fluid output profiles to minimize the confounding effect of fluid output on net fluid balance. Fourth, although further double-blinded, interventional, large, randomized controlled trials are needed to confirm these findings, this multicenter nationwide study has a low selection bias. Finally, we also assessed secondary outcomes including freedom of mechanical ventilation and discharge from the ICU, which we found to be consistent with the 28-day mortality results.

Nevertheless, our current study should also be interpreted in the context of several limitations. First, we may have missed the possible influence of unmeasured factors despite adjustments for confounders. For example, information about the type of fluid or nutritional support was not available. Second, the fluid profiles of our study subjects were collected by day, so we could not analyze the effects of fluid balance over a shorter time frame during the first three days of ICU care. Moreover, the actual duration of D1 might have differed from patient to patient. Third, no data regarding attempts to reduce fluid accumulation were available. For instance, the use of diuretics was not recorded in the database, and the effect of diuretics on prognosis could not be evaluated in this study. Finally, caution is needed in interpreting the results because this study was conducted on a single ethnic group.

## Conclusions

In patients with sepsis, a positive fluid balance on the first ICU day was not associated with mortality; however, a positive fluid balance that continued onto the second or third ICU day was significantly associated with mortality. Particularly, the fluid balance on the second ICU day was associated with the highest risk for mortality in these patients.

### Supplementary Information

**Additional file 1: Figure S1. **Study subject flow chart. ICU, intensive care unit; PS, propensity score. **Figure S2. **Absolute standardized mean differences between negative fluid balance group and positive fluid balance group before and after propensity score matching by the fluid balance of (a) the Day 1, (B) Day 2, and (C) Day 3. The *horizontal axis* represents the standardized mean differences and *red line* indicates the absolute standardized mean difference of 0.1. *Open dots* reflect values prior to matching, and *black dots* after matching. Matching succeeded in reducing the standardized mean difference within an absolute value of 0.1. *SOFA*, sequential organ failure assessment; *CRRT*, continuous renal replacement therapy; *ICU*, intensive care unit. **Table S1. **Baseline Characteristics in the full patients of cohorts with sepsis by the first Day 1-3 of fluid balance. **Table S2. **The profile of fluid input and output in the propensity score matching cohorts by the Day 1-3 of fluid balance.

## Data Availability

The data of this article are available upon reasonable request to the corresponding author.

## Abbreviations

ICU: Intensive care unit
Sepsis-3: The third International Consensus Definitions for Sepsis and Septic Shock
D: Day
SMD: Standardized mean differences
IQR: Interquartile ranges
HR: Hazard ratios
CI: Confidence interval
sHR: Sub-hazard ratios
