# Supplementary material for: Impact of a cumulative positive fluid balance during the first three ICU days in patients with sepsis: a propensity score-matched cohort study
Source: Ann Intensive Care. 2023 Oct 19;13:105. doi: 10.1186/s13613-023-01178-x (PMC10584773; doi:10.1186/s13613-023-01178-x)
Supplement: Supplementary file 1 — Additional file 1: Figure S1. Study subject flow chart. ICU, intensive care unit; PS, propensity score. Figure S2. Absolute standardized mean differences between negative fluid balance group and positive fluid balance group before and after propensity score matching by the fluid balance of (a) the Day 1, (B) Day 2, and (C) Day 3. The horizontal axis represents the standardized mean differences and red line indicates the absolute standardized mean difference of 0.1. Open dots reflect values prior to matching, and black dots after matching. Matching succeeded in reducing the standardized mean difference within an absolute value of 0.1. SOFA, sequential organ failure assessment; CRRT, continuous renal replacement therapy; ICU, intensive care unit. Table S1. Baseline Characteristics in the full patients of cohorts with sepsis by the first Day 1-3 of fluid balance. Table S2. The profile of fluid input and output in the propensity score matching cohorts by the Day 1-3 of fluid balance. [file 13613_2023_1178_MOESM1_ESM.docx]

**Impact of a cumulative positive fluid balance during the first three ICU days in patients with sepsis: a propensity score-matched cohort study**

Dong-gon Hyun, MD, Jee Hwan Ahn, MD, Jin Won Huh, MD, Ph.D., Sang-Bum Hong, MD, Ph.D., Younsuck Koh, MD, Ph.D., FCCM, Dong Kyu Oh, MD, Su Yeon Lee, MD, Mi Hyeon Park, Haein Lee, and Chae-Man Lim, MD, Ph.D., FCCM^*^ on behalf of the Korean Sepsis Alliance (KSA) Investigators

Department of Pulmonary and Critical Care Medicine, Asan Medical Center, University of Ulsan College of Medicine, Seoul, Republic of Korea

*Correspondence to: Chae-Man Lim, M.D., Ph.D., FCCM

Department of Pulmonary and Critical Care Medicine, Asan Medical Center, University of Ulsan College of Medicine, 88 Olympic-ro 43-gil, Songpa-gu, Seoul 05505, Korea

Fax: 82-2-3010-6968

E-mail: [cmlim@amc.seoul.kr](mailto:cmlim@amc.seoul.kr)

**
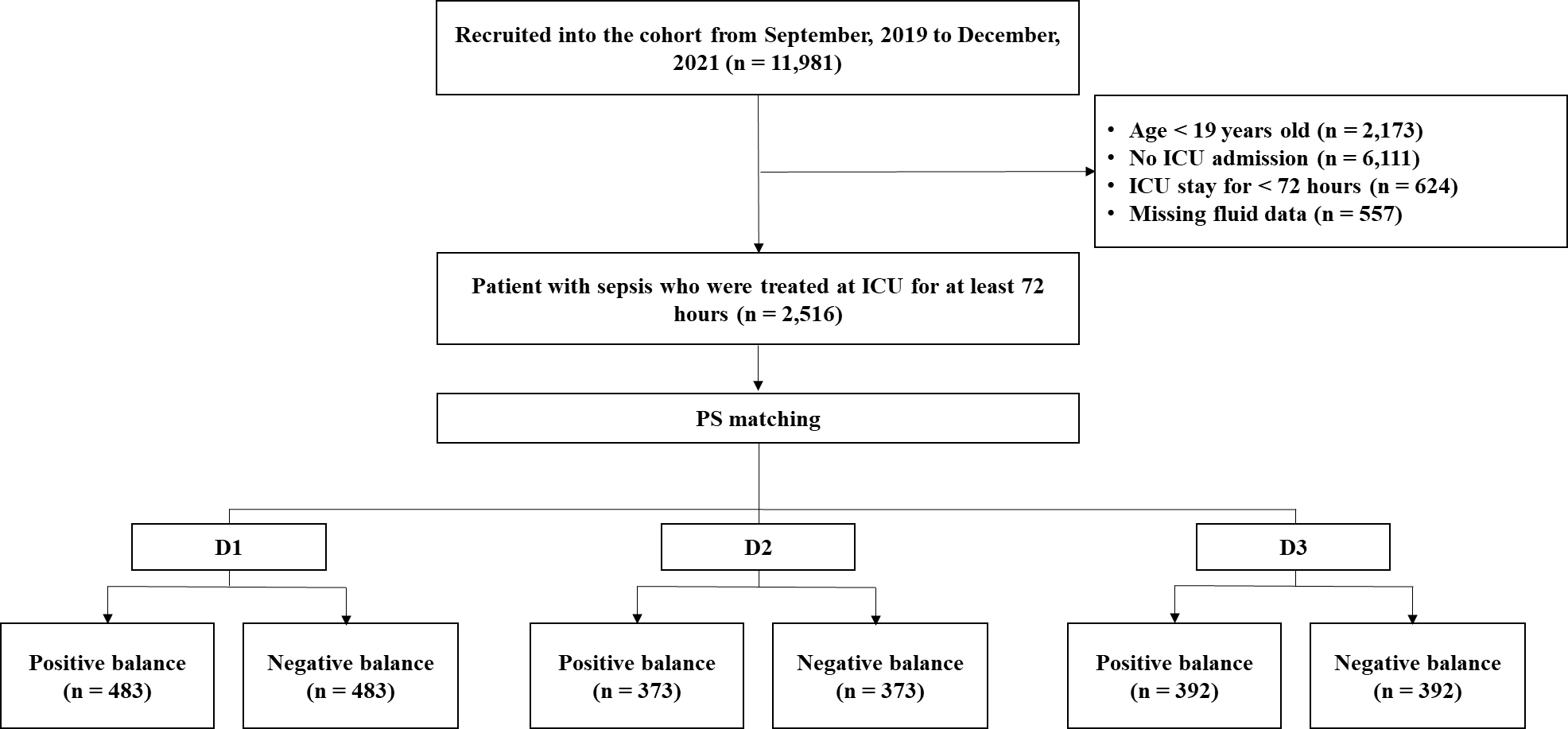
**

**Fig. S1.** Study subject flow chart.

ICU, intensive care unit; PS, propensity score.

**
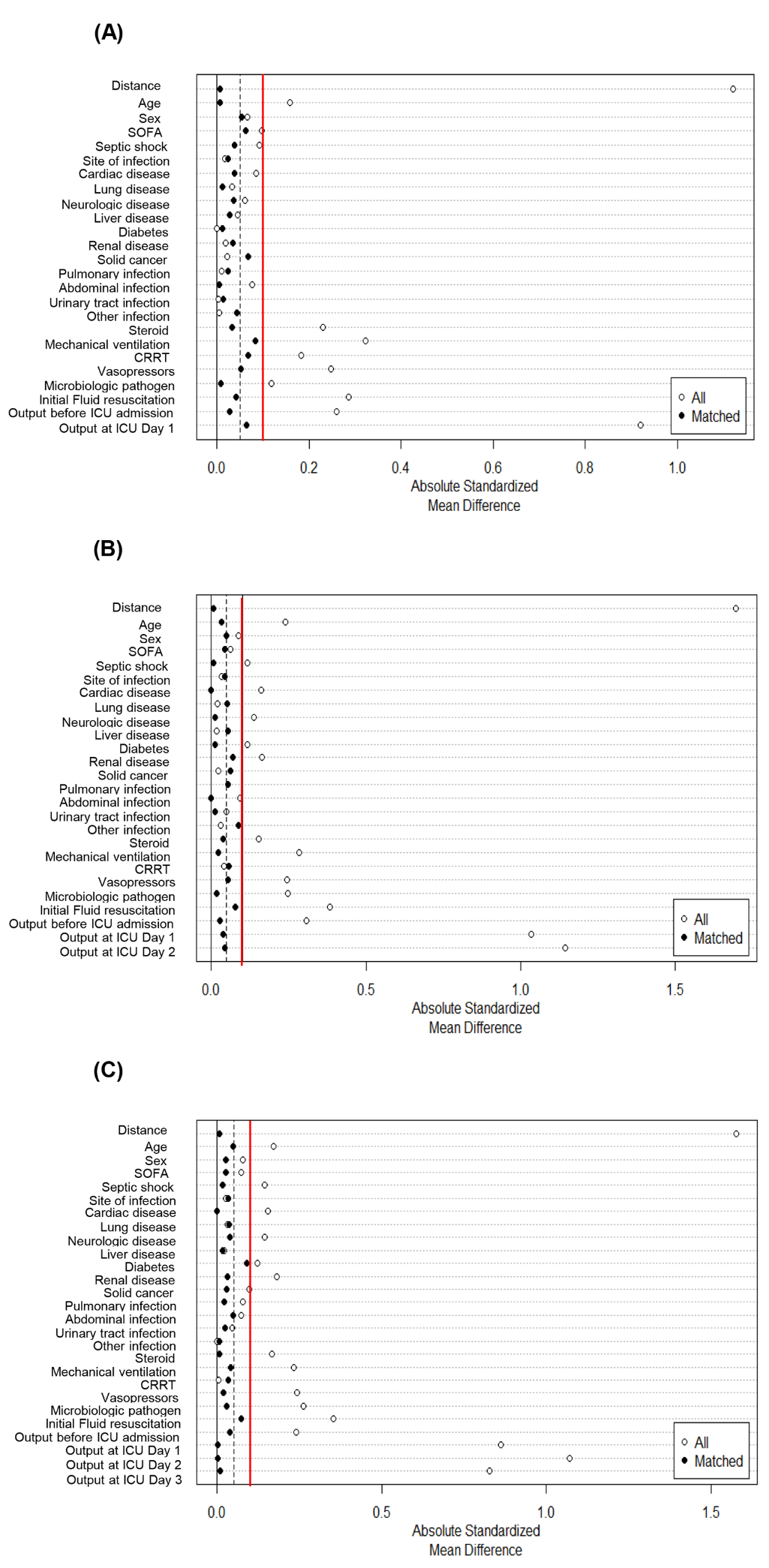
**

**Fig. S2** Absolute standardized mean differences between negative fluid balance group and positive fluid balance group before and after propensity score matching by the fluid balance of (a) the Day 1, (B) Day 2, and (C) Day 3. The *horizontal axis* represents the standardized mean differences and *red line* indicates the absolute standardized mean difference of 0.1. *Open dots* reflect values prior to matching, and *black dots* after matching. Matching succeeded in reducing the standardized mean difference within an absolute value of 0.1.

*SOFA*, sequential organ failure assessment; *CRRT*, continuous renal replacement therapy; *ICU*, intensive care unit.

**Table S1.** Baseline Characteristics in the full patients of cohorts with sepsis by the first Day 1-3 of fluid balance

|  | **Total cohort** | **Day 1 cohort** | |  | **Day 2 cohort** | |  | **Day 3 cohort** | |  |
| --- | --- | --- | --- | --- | --- | --- | --- | --- | --- | --- |
| **Characteristic** | **(n = 2,516)** | **Negative**  **(n = 585)** | **Positive**  **(n = 1931)** | **SMD** | **Negative**  **(n = 499)** | **Positive**  **(n = 2017)** | **SMD** | **Negative**  **(n = 537)** | **Positive**  **(n = 1979)** | **SMD** |
| Female, % | 1066 (42.4) | 233 (39.8) | 833 (43.1) | 0.067 | 194 (38.9) | 872 (43.2) | 0.089 | 211 (39.3) | 855 (43.2) | 0.08 |
| Age, yr | 74.00  [64.00, 81.00] | 72.00  [62.00, 81.00] | 75.00  [65.00, 82.00] | 0.156 | 71.00  [60.00, 80.00] | 75.00  [65.00, 82.00] | 0.23 | 72.00  [61.00, 80.00] | 75.00  [65.00, 82.00] | 0.169 |
| BMI, kg/m^2^ (n = 2,450) | 21.70  [18.94, 24.52] | 22.03  [19.10, 25.11] | 21.54  [18.90, 24.35] | 0.141 | 22.22  [19.72, 25.15] | 21.50  [18.75, 24.42] | 0.213 | 22.04  [19.53, 24.98] | 21.51  [18.75, 24.43] | 0.189 |
| Resuscitative fluid, % | 2091 (83.1) | 441 (75.4) | 1650 (85.4) | 0.256 | 361 (72.3) | 1730 (85.8) | 0.335 | 394 (73.4) | 1697 (85.8) | 0.311 |
| Severity scores |  |  |  |  |  |  |  |  |  |  |
| SOFA score | 7.00  [5.00, 9.00] | 7.00  [5.00, 9.00] | 7.00  [5.00, 9.00] | 0.103 | 7.00  [5.00, 9.00] | 7.00  [5.00, 9.00] | 0.064 | 7.00  [5.00, 8.00] | 7.00  [5.00, 9.00] | 0.074 |
| Septic shock, % | 611 (24.3) | 124 (21.2) | 487 (25.2) | 0.095 | 101 (20.2) | 510 (25.3) | 0.121 | 104 (19.4) | 507 (25.6) | 0.15 |
| Referring facility, % |  |  |  | 0.061 |  |  | 0.24 |  |  | 0.191 |
| Community | 1677 (66.7) | 397 (67.9) | 1280 (66.3) |  | 338 (67.7) | 1339 (66.4) |  | 363 (67.6) | 1314 (66.4) |  |
| Health care | 198 (7.9) | 39 (6.7) | 159 (8.2) |  | 17 (3.4) | 181 (9.0) |  | 23 (4.3) | 175 (8.8) |  |
| Hospital | 641 (25.5) | 149 (25.5) | 492 (25.5) |  | 144 (28.9) | 497 (24.6) |  | 151 (28.1) | 490 (24.8) |  |
| Comorbidities, % |  |  |  |  |  |  |  |  |  |  |
| Cardiac | 643 (25.6) | 166 (28.4) | 477 (24.7) | 0.083 | 155 (31.1) | 488 (24.2) | 0.154 | 165 (30.7) | 478 (24.2) | 0.148 |
| Lung | 377 (15.0) | 93 (15.9) | 284 (14.7) | 0.033 | 72 (14.4) | 305 (15.1) | 0.02 | 85 (15.8) | 292 (14.8) | 0.03 |
| Neurologic | 716 (28.5) | 154 (26.3) | 562 (29.1) | 0.062 | 117 (23.4) | 599 (29.7) | 0.142 | 125 (23.3) | 591 (29.9) | 0.15 |
| Liver | 241 (9.6) | 62 (10.6) | 179 (9.3) | 0.044 | 50 (10.0) | 191 (9.5) | 0.019 | 54 (10.1) | 187 (9.4) | 0.02 |
| Diabetes mellitus | 984 (39.1) | 229 (39.1) | 755 (39.1) | 0.001 | 218 (43.7) | 766 (38.0) | 0.116 | 235 (43.8) | 749 (37.8) | 0.121 |
| Renal disease | 370 (14.7) | 83 (14.2) | 287 (14.9) | 0.019 | 96 (19.2) | 274 (13.6) | 0.153 | 105 (19.6) | 265 (13.4) | 0.167 |
| Connective tissue disease | 67 (2.7) | 14 (2.4) | 53 (2.7) | 0.022 | 16 (3.2) | 51 (2.5) | 0.041 | 15 (2.8) | 52 (2.6) | 0.01 |
| Immunocompromised | 56 (2.2) | 18 (3.1) | 38 (2.0) | 0.071 | 13 (2.6) | 43 (2.1) | 0.031 | 19 (3.5) | 37 (1.9) | 0.103 |
| Hematologic malignancy | 94 (3.7) | 19 (3.2) | 75 (3.9) | 0.034 | 11 (2.2) | 83 (4.1) | 0.109 | 15 (2.8) | 79 (4.0) | 0.066 |
| Solid cancer | 614 (24.4) | 147 (25.1) | 467 (24.2) | 0.022 | 118 (23.6) | 496 (24.6) | 0.022 | 113 (21.0) | 501 (25.3) | 0.101 |
| Site of infection, % |  |  |  |  |  |  |  |  |  |  |
| Respiratory | 1216 (48.3) | 285 (48.7) | 931 (48.2) | 0.01 | 252 (50.5) | 964 (47.8) | 0.054 | 276 (51.4) | 940 (47.5) | 0.078 |
| Abdominal | 610 (24.2) | 127 (21.7) | 483 (25.0) | 0.078 | 105 (21.0) | 505 (25.0) | 0.095 | 117 (21.8) | 493 (24.9) | 0.074 |
| Urinary tract | 586 (23.3) | 137 (23.4) | 449 (23.3) | 0.004 | 108 (21.6) | 478 (23.7) | 0.049 | 117 (21.8) | 469 (23.7) | 0.046 |
| Others**^a^** | 323 (12.8) | 76 (13.0) | 247 (12.8) | 0.006 | 68 (13.6) | 255 (12.6) | 0.029 | 69 (12.8) | 254 (12.8) | <0.001 |
| Laboratory findings |  |  |  |  |  |  |  |  |  |  |
| White blood cell count *10^3^/L | 12.12  [7.65, 17.60] | 12.10  [8.11, 16.70] | 12.18  [7.50, 17.92] | 0.057 | 11.47  [7.70, 16.50] | 12.30  [7.64, 17.93] | 0.101 | 11.74  [7.80, 16.90] | 12.20  [7.51, 17.80] | 0.046 |
| C-reactive protein, mg/dL | 12.30  [3.68, 22.06] | 11.43  [3.10, 20.49] | 12.70  [3.87, 22.36] | 0.086 | 11.32  [2.44, 20.78] | 12.70  [3.96, 22.19] | 0.082 | 11.64  [2.60, 20.87] | 12.52  [3.96, 22.20] | 0.075 |
| Procalcitonin (n = 1,356),  ng/mL | 6.02  [0.77, 35.42] | 3.48  [0.34, 34.82] | 6.58  [0.93, 35.47] | 0.069 | 2.70  [0.34, 26.18] | 6.76  [0.93, 37.36] | 0.092 | 4.78  [0.39, 28.49] | 6.37  [0.90, 37.43] | 0.081 |
| Lactic acid (n = 2,483),  mmol/L | 3.00  [1.77, 5.50] | 2.68  [1.50, 4.60] | 3.20  [1.90, 5.77] | 0.224 | 2.63  [1.50, 4.59] | 3.20  [1.82, 5.70] | 0.218 | 2.60  [1.50, 4.47] | 3.20  [1.90, 5.73] | 0.256 |
| Adjunct interventions, % |  |  |  |  |  |  |  |  |  |  |
| Steroids | 561 (22.3) | 86 (14.7) | 475 (24.6) | 0.251 | 85 (17.0) | 476 (23.6) | 0.164 | 90 (16.8) | 471 (23.8) | 0.176 |
| Mechanical ventilation | 1274 (50.6) | 224 (38.3) | 1050 (54.4) | 0.327 | 196 (39.3) | 1078 (53.4) | 0.287 | 223 (41.5) | 1051 (53.1) | 0.234 |
| CRRT | 430 (17.1) | 68 (11.6) | 362 (18.7) | 0.199 | 79 (15.8) | 351 (17.4) | 0.042 | 91 (16.9) | 339 (17.1) | 0.005 |
| ECMO | 20 ( 0.8) | 5 ( 0.9) | 15 (0.8) | 0.009 | 6 (1.2) | 14 (0.7) | 0.052 | 4 (0.7) | 16 (0.8) | 0.007 |
| Vasopressors | 1962 (78.0) | 412 (70.4) | 1550 (80.3) | 0.23 | 350 (70.1) | 1612 (79.9) | 0.227 | 378 (70.4) | 1584 (80.0) | 0.225 |
| Microbiologic pathogen, % | 1665 (66.2) | 362 (61.9) | 1303 (67.5) | 0.117 | 284 (56.9) | 1381 (68.5) | 0.241 | 304 (56.6) | 1361 (68.8) | 0.253 |
| Bacteria | 1574 (94.5) | 344 (95.0) | 1230 (94.4) | 0.028 | 267 (94.0) | 1307 (94.6) | 0.027 | 289 (95.1) | 1285 (94.4) | 0.029 |
| Virus | 47 (2.8) | 11 (3.0) | 36 (2.8) | 0.016 | 10 (3.5) | 37 (2.7) | 0.049 | 9 (3.0) | 38 (2.8) | 0.01 |
| Fungus | 90 (5.4) | 16 (4.4) | 74 (5.7) | 0.058 | 16 (5.6) | 74 (5.4) | 0.012 | 15 (4.9) | 75 (5.5) | 0.026 |
| MDR (n = 1458) | 675 (46.3) | 157 (48.6) | 518 (45.6) | 0.06 | 113 (44.7) | 562 (46.6) | 0.039 | 115 (42.8) | 560 (47.1) | 0.087 |

*BMI*, body mass index; *SOFA*, sequential organ failure assessment; *CRRT*, continuous renal replacement therapy, *ECMO*, extracorporeal membrane oxygenation; *MDR*, multi-drug resistance

**^a^**Others included skin/soft tissue infection, catheter associated infection, neurologic infection and unknown.

**Table S2.** The profile of fluid input and output in the propensity score matching cohorts by the Day 1-3 of fluid balance

|  | **Total cohort** | **Day 1 cohort (n = 966)** | |  | **Day 2 cohort (n = 746)** | |  | **Day 3 cohort (n = 784)** | |  |
| --- | --- | --- | --- | --- | --- | --- | --- | --- | --- | --- |
|  | **(n = 2,516)** | **Negative**  **(n = 483)** | **Positive**  **(n = 483)** | **P** | **Negative**  **(n = 373)** | **Positive**  **(n = 373)** | **P** | **Negative**  **(n = 392)** | **Positive**  **(n = 392)** | **P** |
| Before ICU admission, mL | | | | | | | | | |  |
| Input | 1892.25  [1050.00, 2803.98] | 1881.00  [976.00, 2854.45] | 1720.00  [1000.00, 2750.00] | 0.151 | 1702.00  [893.00, 2830.00] | 1731.00  [1000.00, 2763.00] | 0.274 | 1636.00  [849.50, 2681.28] | 1749.00  [1047.60, 2900.00] | 0.169 |
| Output | 400.00  [140.00, 916.25] | 620.00  [277.50, 1250.00] | 500.00  [170.00, 1056.50] | 0.700 | 603.00  [212.00, 1225.00] | 545.00  [200.00, 1160.00] | 0.779 | 600.00  [200.00, 1290.75] | 600.00  [230.00, 1200.00] | 0.643 |
| Balance | 1279.65  [468.80, 2220.00] | 1032.00  [194.00, 2010.50] | 1028.00  [380.00, 2003.40] | 0.136 | 902.00  [143.00, 1861.90] | 940.00  [234.60, 2050.00] | 0.256 | 900.00  [126.15, 1923.25] | 1001.25  [300.00, 1983.28] | 0.002 |
| ICU Day 1. mL | | | | | | | | | |  |
| Input | 1304.30  [619.22, 2449.00] | 640.00  [280.05, 1204.60] | 2003.60  [1021.65, 3114.00] | <0.001 | 849.60  [411.20, 1640.00] | 2025.00  [1019.00, 3312.00] | <0.001 | 869.10  [420.00, 1695.50] | 1907.30  [885.08, 3208.00] | <0.001 |
| Output | 675.00  [275.00, 1417.00] | 970.00  [505.00, 1754.00] | 919.00  [412.50, 1741.00] | 0.376 | 1175.00  [555.00, 2034.00] | 1150.00  [500.00, 2055.00] | 0.701 | 1112.00  [470.00, 1922.50] | 1042.50  [437.50, 1853.75] | 0.982 |
| Balance | 449.25  [26.33, 1160.10] | -270.00  [-538.10, -94.00] | 795.00  [314.15, 1579.00] | <0.001 | -224.00 [-626.30  80.00] | 750.00  [186.00, 1507.70] | <0.001 | -80.95  [-490.58, 195.75] | 599.05  [160.00, 1511.88] | <0.001 |
| ICU Day 2, mL | | | | | | | | | |  |
| Input | 2998.40  [2311.28, 3870.70] | 2588.50  [2004.25, 3336.50] | 3000.00  [2370.50, 3879.40] | <0.001 | 2229.90  [1782.50, 2826.00] | 3472.00  [2770.00, 4372.00] | <0.001 | 2237.60  [1759.83, 2835.07] | 3569.50  [2854.25, 4321.80] | <0.001 |
| Output | 2040.00  [1297.25, 2969.50] | 2428.00  [1762.50, 3272.50] | 2130.00  [1467.50, 3094.50] | 0.003 | 2860.00  [2280.00, 3550.00] | 2830.00  [1990.00, 3645.00] | 0.561 | 2720.00  [2148.75, 3467.50] | 2850.00  [2078.75, 3646.25] | 0.982 |
| Balance | 848.65  [39.98, 1757.08] | 160.00  [-573.50, 843.50] | 762.10  [58.50, 1564.40] | <0.001 | -527.00  [-1127.00, -57.00] | 636.00  [124.30, 1357.00] | <0.001 | -441.00  [-1034.00, 43.67] | 649.95  [101.00, 1366.20] | <0.001 |
| ICU Day 3, mL | | | | | | | | | |  |
| Input | 2577.20  [1940.53, 3357.48] | 2377.60  [1800.50, 3077.40] | 2578.00  [1913.00, 3403.90] | 0.002 | 2164.00  [1584.50, 2805.00] | 3070.00  [2308.00, 3820.00] | <0.001 | 2016.45  [1519.50, 2599.00] | 3113.50  [2342.25, 3941.75] | <0.001 |
| Output | 2080.00  [1270.00, 2970.00] | 2325.00  [1537.50, 3137.50] | 2155.00  [1375.00, 3027.50] | 0.114 | 2300.00  [1645.00, 3140.00] | 2510.00  [1700.00, 3550.00] | 0.005 | 2591.50  [1945.25, 3345.50] | 2675.00  [1799.75, 3613.75] | 0.902 |
| Balance | 415.00  [-278.00, 1187.25] | 640.00  [280.05, 1204.60] | 2003.60  [1021.65, 3114.00] | <0.001 | -165.00  [-681.70, 398.00] | 408.60  [-270.00, 1122.00] | <0.001 | -473.00  [-1141.28, -35.75] | 419.50  [-162.00, 1015.68] | <0.001 |
| Cumulative balance, mL | | | | | | | | | |  |
| Day 1 | 449.25  [26.33, 1160.10] | -270.00  [-538.10, -94.00] | 795.00  [314.15, 1579.00] | <0.001 | -224.00  [-626.30, 80.00] | 750.00  [186.00, 1507.70] | <0.001 | -80.95  [-490.58, 195.75] | 599.05  [160.00, 1511.88] | <0.001 |
| Day 2 | 1444.96  [273.00, 2840.25] | -162.80  [-1096.90, 632.05] | 1702.80  [821.50, 2806.40] | <0.001 | -662.00  [-1333.10, -308.00] | 1470.00  [734.80, 2457.00] | <0.001 | -543.50  [-1256.65, -10.00] | 1479.40  [662.60, 2603.00] | <0.001 |
| Day 3 | 1927.80  [298.42, 3835.90] | -74.00  [-1502.50, 1068.50] | 2137.30  [792.10, 3795.65] | <0.001 | -907.40  [-1952.00, -207.00] | 2017.30  [885.70, 3330.00] | <0.001 | -1001.30  [-1826.85, -441.05] | 1778.35  [848.25, 3250.25] | <0.001 |

*ICU* intensive care unit

**Table S3.** Hazard ratios of death at day 28 according to the amount of fluid balance for each cohort

|  | **Day 1 cohort (n = 966)** | |  | **Day 2 cohort (n = 746)** | |  | **Day 3 cohort (n = 784)** | |  |
| --- | --- | --- | --- | --- | --- | --- | --- | --- | --- |
| **Variable** | **HR** | **95% CI** | **P** | **HR** | **95% CI** | **P** | **HR** | **95% CI** | **P** |
| Amount of fluid balance | Day 1 | | | Day 2 | | | Day 3 | | |
| Fluid balance < 0 L | Reference |  |  | Reference |  |  | Reference |  |  |
| 0 L ≤ Fluid balance < 1 L | 1.122 | 0.767-1.641 | 0.553 | 1.483 | 0.871-2.526 | 0.147 | 0.907 | 0.501-1.640 | 0.746 |
| 1 L ≤ Fluid balance < 2 L | 1.347 | 0.832-2.180 | 0.226 | 1.985 | 1.217-3.237 | 0.006 | 1.650 | 0.983-2.773 | 0.058 |
| 2 L ≤ Fluid balance | 1.060 | 0.585-1.920 | 0.848 | 2.818 | 1.837-4.323 | < 0.001 | 1.932 | 1.297-2.878 | 0.001 |

*HR*, hazard ratio; *CI*, confidence interval.
